# Peptidome and Transcriptome Analysis of Plant Peptides Involved in *Bipolaris maydis* Infection of Maize

**DOI:** 10.3390/plants12061307

**Published:** 2023-03-14

**Authors:** Pijie Sheng, Minyan Xu, Zhenzhen Zheng, Xiaojing Liu, Wanlu Ma, Ting Ding, Chenchen Zhang, Meng Chen, Mengting Zhang, Beijiu Cheng, Xin Zhang

**Affiliations:** 1 National Engineering Laboratory of Crop Stress Resistance Breeding, School of Life Sciences, Anhui Agricultural University, Hefei 230036, China; 2 Key Laboratory of Crop Biology of Anhui Province, School of Life Sciences, Anhui Agricultural University, Hefei 230036, China; 3 School of Plant Protection, Anhui Agricultural University, Hefei 230036, China

**Keywords:** *Zea mays* L., *Bipolaris maydis*, southern corn leaf blight, transcriptomics, peptidomics

## Abstract

Southern corn leaf blight (SCLB) caused by *Bipolaris maydis* threatens maize growth and yield worldwide. In this study, TMT-labeled comparative peptidomic analysis was established between infected and uninfected maize leaf samples using liquid-chromatography-coupled tandem mass spectrometry. The results were further compared and integrated with transcriptome data under the same experimental conditions. Plant peptidomic analysis identified 455 and 502 differentially expressed peptides (DEPs) in infected maize leaves on day 1 and day 5, respectively. A total of 262 common DEPs were identified in both cases. Bioinformatic analysis indicated that the precursor proteins of DEPs are associated with many pathways generated by SCLB-induced pathological changes. The expression profiles of plant peptides and genes in maize plants were considerably altered after *B. maydis* infection. These findings provide new insights into the molecular mechanisms of SCLB pathogenesis and offer a basis for the development of maize genotypes with SCLB resistance.

## 1. Introduction

Maize (*Zea mays* L.) is the most productive food and energy crop worldwide. Maize is susceptible to biotic and abiotic stresses that result in growth changes, as well as reductions in quality and yield [1,2]. Common maize diseases caused by various parasitic and semiparasitic pathogens include maize stalk rot, corn rust, corn smut, northern corn leaf blight, southern corn leaf blight, etc. [3,4]. The incidence and prevalence of these diseases varies with maize germplasm, pathogen type, planting region, and season [5]. Foliar diseases caused by fungal pathogens are a major threat to maize growth [3,4]. Pathogenesis is mainly associated with a reduction in photosynthetic area, chlorosis, and premature leaf senescence [6].

Southern corn leaf blight (SCLB) caused by *Bipolaris maydis* (*B. maydis*) is a typical wind-borne foliar fungal disease that is difficult to control and spreads widely worldwide, especially in warm and humid regions, such as the Southeastern United States or summer corn-growing areas along the Yangtze River and Huai River basins of China. This pathogen has two races (race O and race T) [7] and was initially described as a mild pathogen in 1925; however, in 1970 and 1971, the wide spread of the race T pathogen in the U.S. Corn Belt, especially in male sterile cytoplasmic corn in Texas [8], caused a massive SCLB epidemic, resulting in 15% yield reduction and an estimated USD one-billion loss nationwide. Since then, *B. maydis* has been recognized as an important pathogen and SCLB has become one of the most destructive foliar diseases. Studies have shown that HmT toxin molecule produced by this pathogen promotes the expansion of mitochondria, resulting in abnormal oxidative phosphorylation and mitochondrial respiration and ultimately leading to cell death and maize pathogenesis, suggesting that the reaction between HmT toxin and mitochondria is crucial for the development of SCLB disease [9]. Most studies on SCLB focus on quantitative trait loci (QTL) for resistance to this disease and dozens of QTLs have been identified to be significantly associated with the disease [10,11,12,13]. In recent years, some biological control methods and extracts have shown antagonistic activity against *B. maydis*, including a cyclic lipopeptide antibiotic (iturin A2) purified from *Bacillus subtilis* B47 [14], the metabolites of *Bacillus cereus* C1L [15], and *B. subtilis* dzsy21 and its lipopeptides [16].

Peptides differ from proteins by the amount of amino acid residues the molecule contains. Traditionally, peptides are defined as molecules that consist of between 2 and 50 amino acids, whereas proteins are made up of 50 or more amino acids. In addition, peptides tend to be less well defined in terms of structure than proteins. Peptides are found in humans, animals, plants, and microorganisms. Many plant peptides have been studied and shown to play diverse and important roles and functions in plant growth and development [17,18,19,20], intercellular signaling [21], stress-signaling molecules [22,23,24], innate immune responses [25,26], and regulation of nutrient transport and utilization [27]. Peptidomics is a branch of proteomics that can be employed to identify and verify all endogenous peptides in biological samples, as well as to compare expression levels of target peptides in specific biochemical processes to provide sufficient data to study the structure and function of peptides. So far, peptidomics studies have mostly focused on neuropeptides and hormones in human diseases [28,29,30,31,32,33,34], and only a few studies have been conducted on plant peptidomics, including a database of unannotated secreted peptides in *Arabidopsis* [35], the role of wound-induced peptides in tomato [36], diversity analysis of cyclotides from *Viola tricolor* [37], and the role of an apoplastic peptide activates salicylic acid signaling in maize [38].

In the present study, a comparative peptidomics profile was generated using liquid chromatography–tandem mass spectrometry and integrated with the transcriptome data previously obtained under identical experimental conditions to investigate the role of plant peptides in response to *B. maydis* infection in maize.

## 2. Results

### 2.1. Identification of Phenotypes and Characteristics of SCLB-Infected Maize

**Figure 1:**
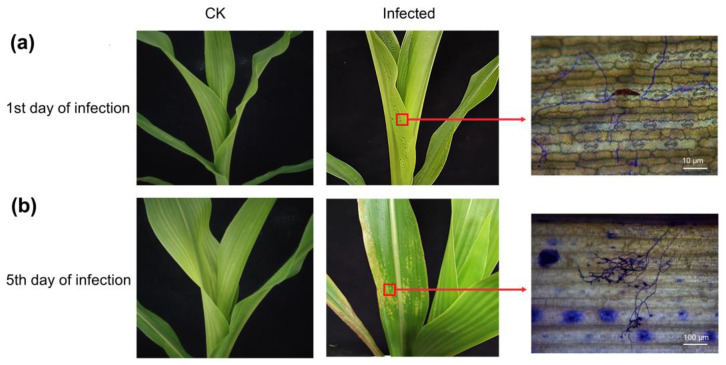
The phenotype of maize leaves after *Bipolaris maydis* infection on (**a**) day 1 and (**b**) day 5. The insets indicated by arrows show the corresponding conidia and mycelium growth observed under the microscope.

Compared to the uninfected control group, disease symptoms, especially lesions, gradually appeared on maize leaves inoculated with *B. maydis* spores. After infection, symptom manifestation started with the appearance of light-gray translucent water stains on the leaves, accompanied by chlorosis on day 1 (Figure 1a). On day 5, the spots spread rapidly and fused into larger yellowish-brown irregular lesions on the leaves (Figure 1b). Microscopy observation of the infected leaves after trypan blue staining revealed that, on day 1 after infection, conidia of the pathogen began to germinate on the leaves and growing hyphae began to spread. On day 5, dense hyphae proliferated, and oval sporangia gradually appeared on the top of some hyphae (as shown by the arrows).

**Figure 2:**
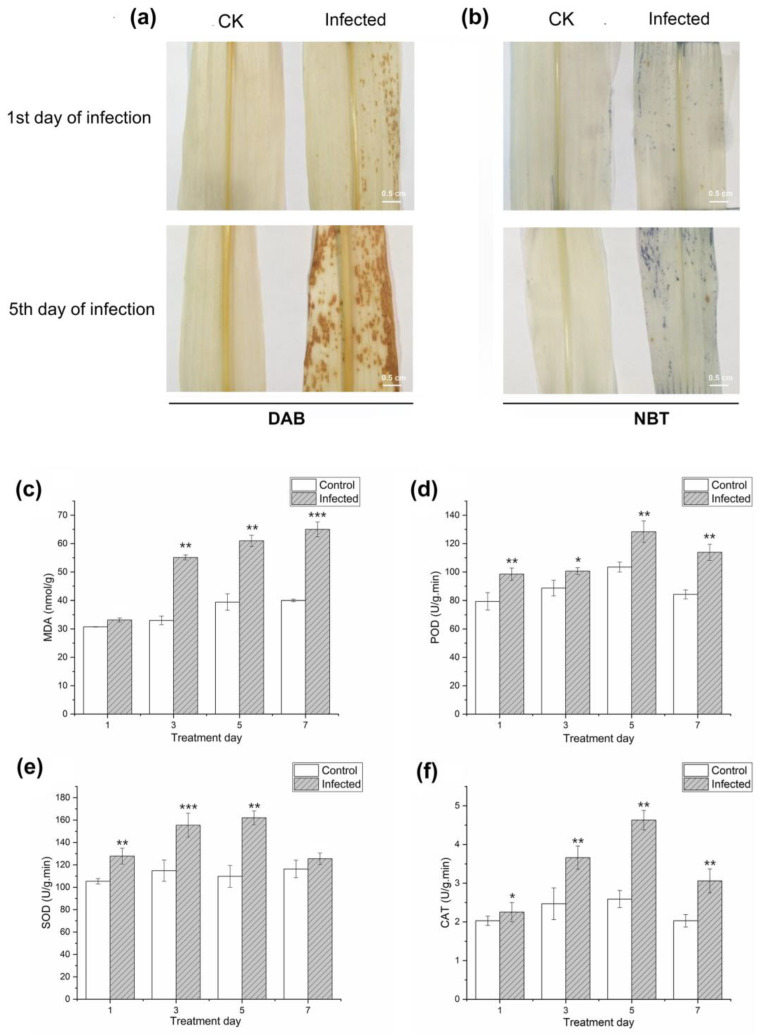
Detection of reactive oxygen species (ROS) accumulation by (**a**) DAB and (**b**) NBT staining in maize leaves and physiological investigation in response to *Bipolaris maydis* infection, including (**c**) MDA content, (**d**) POD activity, (**e**) SOD activity, and (**f**) CAT activity. (Student’s *t*-test, * *p* < 0.05; ** *p* < 0.01; *** *p* < 0.001.).

Pathogen infection induces the accumulation of reactive oxygen species (ROS) in plants. As signaling molecules, ROS trigger a series of resistance cascade responses in plants. To understand the relationship between plant disease resistance and ROS burst, in this study, we performed diaminobenzidine tetrahydrochloride (DAB) and nitrotetrazolium blue chloride (NBT) staining to access the levels of H_2_O_2_ and superoxide ions (O_2_^−^) in the leaves of the infected plants. The distribution and severity of reddish-brown precipitates on the leaves indicate that the accumulation of H_2_O_2_ in the treatment group increased with the aggravation of the disease (Figure 2a). NBT staining showed that the infected maize leaves exhibit dark blue spots that increase in number and intensity as the disease progresses (Figure 2b), indicating the presence of superoxide ions. Studies on ROS accumulation in plants showed that lower concentrations of hydrogen peroxide act as signaling molecules in stress-signal transduction pathways to induce plant defense responses, and a higher concentration of hydrogen peroxide could directly kill invading pathogens. However, excessive hydrogen peroxide can over-oxidize membrane lipids and damage membrane systems and biological macromolecules, such as proteins, lipids, and nucleic acids. Therefore, there should be a balance between the production and scavenging of H_2_O_2_ during plant defense responses.

The activities of superoxide dismutase (SOD), peroxidase (POD), catalase (CAT), and the content of malondialdehyde (MDA) in the infected and uninfected maize treatment groups were measured to determine their physiological changes during plant response to the infection (Figure 2c–f). SOD, POD, and CAT are antioxidant enzymes in plants. In this study, the activities of the three enzymes were higher in the infected maize plants than in the control plants seven days post-infection. The enzyme level in the infected plants increased in the first five days but decreased on the seventh day. This can be attributed to the high consumption of the three enzymes during the elimination of excess free radicals as the disease progressed. Additionally, it may be due to the compensatory effect between different enzymes during disease progression. MDA is the product of membrane lipid peroxidation. The MDA content in infected plants was higher than that in control plants at seven days after infection and increased with time, indicating a positive correlation between MDA content of maize leaves and degree of plant stress.

### 2.2. Illumina Sequencing and DEGs Analysis

Four cDNA libraries were constructed from total RNA extracted from infected maize leaves to identify the genes linked to *B. maydis* infection in maize. Table 1 provides an overview of the RNA-Seq reads generated from four libraries. A higher Q20 value (98.17% and 98.30% in CK and *B. maydis* treatments, respectively) and Q30 value (94.65% and 94.99% in CK and *B. maydis* treatments, respectively) indicated higher transcriptome sequencing quality.

**Figure 3:**
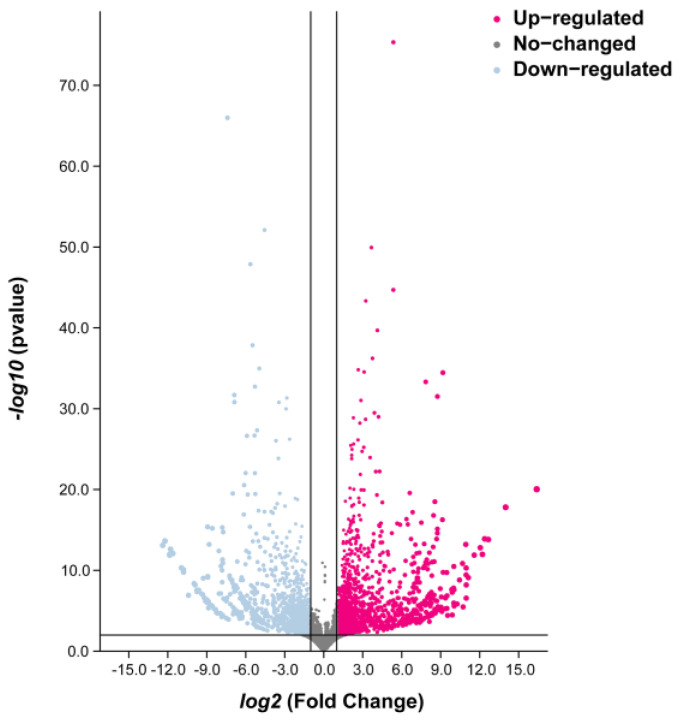
Volcano plot of differential expression genes (DEGs) in the leaves of *Bipolaris maydis* infected maize plants compared to the control group. Red dots denote up-regulated DEGs, light blue dots denote down-regulated DEGs, and gray dots represent genes with no significant difference.

Fragments per kilobase of transcript per million mapped reads (FPKM) was used to determine the gene expression level in the samples and compare the mRNA level of maize in response to SCLB disease. Differentially expressed genes (DEGs) were significantly screened across the different samples based on the criteria of |*log_2_* fold change| ≥2 and *p* ≤ 0.01. Compared with the control group, 2146 DEGs were identified (Table S1), of which 1291 were up-regulated and 856 were down-regulated (Figure 3).

### 2.3. Functional Classification of DEGs

**Figure 4:**
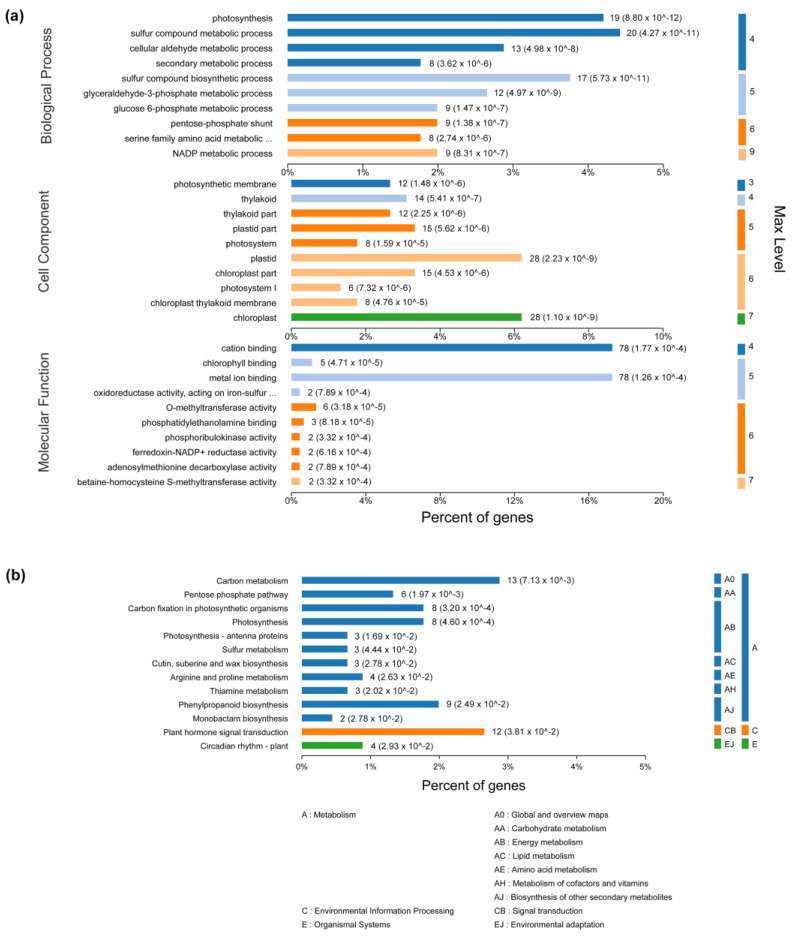
Enrichment analysis of differentially expressed genes (DEGs). (**a**) The results are summarized in three main categories: biological process; cellular component; and molecular function. The *x*-axis indicates the number and percent of genes in each category. The *y*-axis indicates the most enriched GO terms in the three main categories. Max level indicates maximal annotated level of the term in the GO graph. (**b**) KEGG enrichment analysis of 2146 DEGs. The *x*-axis indicates the percent of genes, and the *y*-axis indicates pathway.

To understand the main biological functions of the DEGs in SCLB-infected maize, we performed GO enrichment analysis to establish the primary metabolic processes and signal transduction pathways involved in DEGs. Sulfur compound metabolic process (GO:0006790) and photosynthesis (GO:0015979); plastid (GO:0009536) and chloroplast (GO:0009507); and cation binding (GO:0043169) and metal ion binding (GO:0046872) were the most abundant GO terms in the biological process, cellular component, and molecular function, respectively (Figure 4a). The number of DEGs involved in each KEGG pathway was also investigated to understand the biological and signal transduction pathways in SCLB-infected maize (Figure 4b). Out of 2146 DEGs, 90 significant KEGG pathways (FDR < 5%) were enriched, including carbon metabolism, plant hormone signal transduction, phenylpropanoid biosynthesis, carbon fixation in photosynthetic organisms, photosynthesis, and plant circadian rhythm. Carbon metabolism was the pathway category with the largest number of genes (359), followed by the plant hormone signal transduction pathway.

### 2.4. Identification, Comparison, and Characterization of DEPs

Proteome Discover (ThermoFisher version 2.1) was used for peptide data analyses. A total of 768 peptides were detected in Sample S1, of which 724 were unique peptides belonging to 293 precursor proteins (Table S2). Correspondingly, 712 peptides were detected in Sample S2, of which 672 were unique peptides belonging to 282 precursor proteins (Table S3); meanwhile, 351 peptides were detected in Sample S3, of which 337 were unique peptides belonging to 182 precursor proteins (Table S4). Most peptides (94.5%, 94.7%, and 96.7%) corresponded to the individual precursor protein, revealing that the identified peptides had good sequence coverage and specificity.

**Figure 5:**
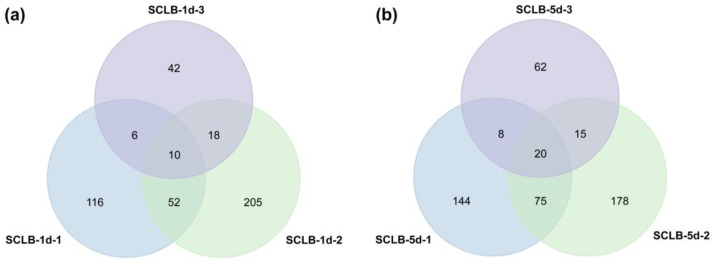
Venn diagrams showing the numbers of differentially expressed peptides (DEPs). (**a**) Overlap of DEPs between SCLB-1d-1, SCLB-1d-2, and SCLB-1d-3. (**b**) Overlap of DEPs between SCLB-5d-1, SCLB-5d-2, and SCLB-5d-3.

By filtering and screening differentially expressed peptides (DEPs) according to the threshold value set by fold change > 1.25 (up-regulated) or < 0.8(down-regulated), 455 and 502 significant DEPs were identified in SCLB-1d and SCLB-5d, respectively, while 262 were common in both cases (Tables S5–S7). A Venn diagram showed that 10 DEPs were common in SCLB-1d-1, SCLB-1d-2, and SCLB-1d-3 (Table 2; Figure 5a), while 20 were common in SCLB-5d-1, SCLB-5d-2, and SCLB-5d-3 (Table 3; Figure 5b).

**Figure 6:**
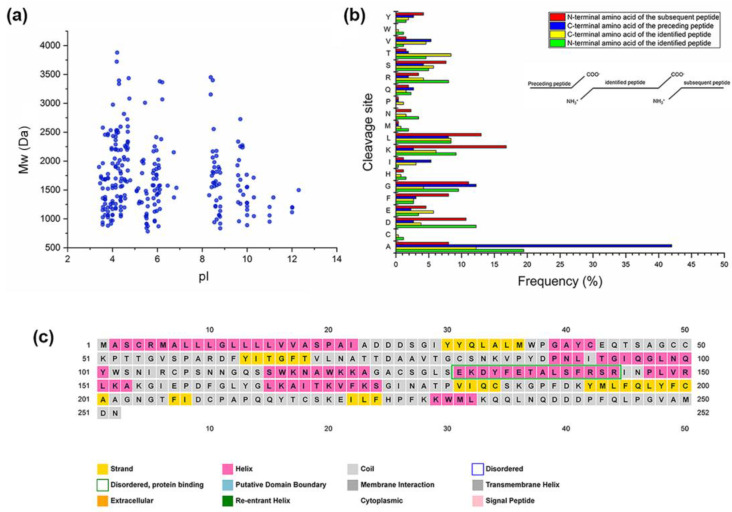
(**a**) Scatter plot of molecular weight (Mw) versus isoelectric point (pI) distribution of common differentially expressed peptides (DEPs); (**b**) histogram showing the cleavage sites of N-terminal and C-terminal amino acid of the preceding and identified peptides; and (**c**) prediction of the secondary structure of precursor protein (B6SSH9) of peptides.

The 262 DEPs were also analyzed based on other parameters, including molecular weight (Mw) and isoelectric point (pI). The results showed that Mw of DEPs mainly varied between 800 and 2500 Da, whereas pI was mainly distributed in the ranges of 3–6 and 8–11. There was a pattern in the relative distribution of Mw versus pI of these peptides, and Mw was mainly gathered into four groups and located around pI 4, 6, 9, and 10 (Figure 6a). Furthermore, we counted the C-terminal and N-terminal amino acids of the preceding peptides to explore the cleavage sites of protease during peptide formation (Figure 6b). It was found that amino acids of the identified peptides occurred in the following sequence: N-terminal sequence A, D, G, and K; C-terminal sequence A, T, and L. The dominant C-terminal amino acids of the preceding peptides were A and G, and most frequent cleavage sites of N-terminal amino acids of the subsequent peptides were K, L, G, and D.

### 2.5. Functional Classification of DEPs’ Precursor Proteins

**Figure 7:**
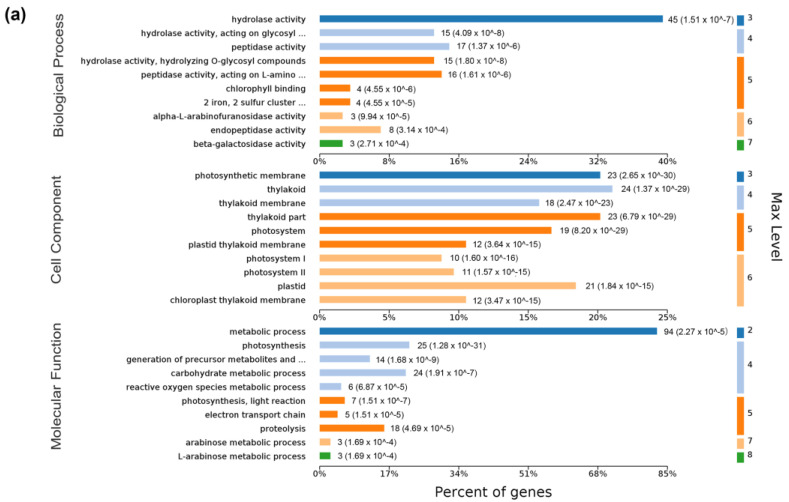

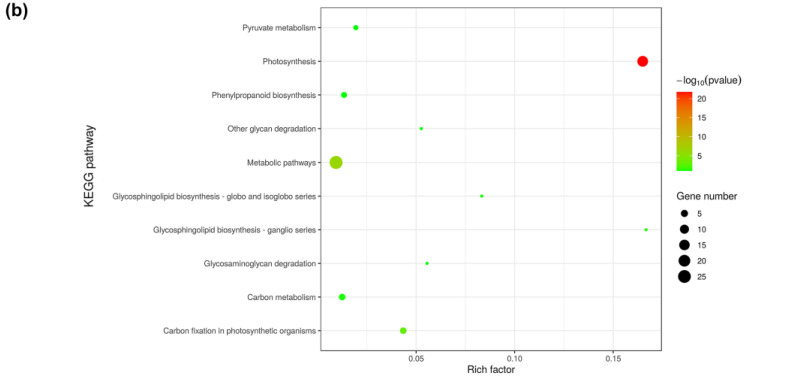
Gene ontology (GO) classification and Kyoto Encyclopedia of Genes and Genomes (KEGG) enrichment of differentially expressed peptides (DEPs) in maize. (**a**) The results are summarized in three main categories: biological process; cellular component; and molecular function. The *x*-axis indicates the number and percent of genes in a category. The *y*-axis indicates the most enriched GO terms in three main categories. Max level indicates maximal annotated level of this term in the GO graph. (**b**) Distribution of enriched KEGG pathway analysis of the 147 precursor proteins of common DEPs. The *x*-axis indicates rich factor and the *y*-axis indicates pathway.

Analysis of the 262 DEPs’ precursor proteins showed that they belong to 147 non-repetitive proteins. GO and pathway enrichment analysis of their precursor proteins suggested that 236 DEPs might have biological events (Figure 7a). GO analysis showed that the main biological process categories were hydrolase activity, peptidase activity, and peptidase activity acting on L-amino acid peptides. The main cellular components involved were thylakoid, thylakoid part, photosynthetic membrane, and plastid. The molecular function levels were mainly related to the metabolic process, photosynthesis, and carbohydrate metabolic process. Enrichment of the KEGG pathway involved two major pathways, namely the photosynthesis and metabolic pathways, accompanied by other pathways, including carbon fixation in photosynthetic organisms, carbon metabolism, phenylpropanoid biosynthesis, pyruvate metabolism, glycosphingolipid biosynthesis in ganglio series and in globo and isoglobo series (Figure 7b).

## 3. Discussion

In natural ecosystems, plants fall victims to biotic and abiotic stresses, which affect their growth, development, yield, and quality [39,40,41,42]. As a result, plants have evolved complex mechanisms to perceive external signals and to respond promptly and adequately to potential phytopathogens [43,44,45,46]. Peptidomics has been extensively applied in the discovery and functional research of neuropeptides and hormones [29,30]. However, there are only a few descriptions of plant biotic stress responses from the perspective of plant peptides and plant peptidomics [47]. In this exemplary study, we searched for and identified many peptide changes in maize peptidome following *B. maydis* infection. Subsequently, we summarized the adaptive or defensive peptides produced by maize in response to SCLB stress. The knowledge gathered from the characteristics of the DEPs in infected maize helps us further understand the pathophysiology of SCLB development and provides an effective technique for improving disease prevention and treatment. To our knowledge, this is the first study that combines transcriptome and peptidome data to elucidate responses triggered by biostimuli at the plant molecular level. Our results suggest that SCLB disease alters the transcriptome and peptidome of maize, as demonstrated by several DEGs and DEPs.

ROS are key signaling molecules that enable cells to respond rapidly to different stimuli. In plants, ROS play a crucial role in sensing abiotic and biotic stresses, integrating different environmental signals, and activating stress-response networks, which in turn contribute to the establishment of defense mechanisms and plant resilience [48]. In this study, we identified that peroxidase C0PKS1 and cysteine protease 2 B4F9B5 are commonly found in DEGs and DEPs. Cysteine proteases have previously been shown to modulate immunity in maize [49]. In addition to these precursor proteins that were found to be annotated, we identified a number of proteins that are not annotated but frequently appear in peptidomic data (B6TA80, B4FTI5, B6TEI9, etc.), and we suggest that these proteins might perform certain functions through their degradation peptides in response to SCLB stress.

Studies postulate that many biotic defense responses in multicellular organisms are mediated by proteins that act as signal molecules or antimicrobial agents [50,51]. As small molecule proteins, bioactive peptides are also shown to be involved in the defense stress response of organisms [36,38]. In the present study, a comparative peptidome analysis was performed using the TMT-labeling approach to characterize the peptidome of maize infected with *B. maydis* infection, and the results show that *B. maydis* infection significantly alters the peptidome of the stressed plants. A total of 262 DEPs derived from 147 precursor proteins were identified from the peptidome of the infected maize group. Among the DEPs, 10 and 20 common DEPs found in maize leaves on day 1 and day 5 after infection, respectively. Thus, we hypothesized that as the disease progress, maize plants employ various mechanisms to increase their resistance or adaptability, and these mechanisms might involve many peptides associated with defense or growth maintenance. Up- or down-regulated peptides might be related to the tolerance or resistance of maize to SCLB. Of course, further studies on gene silencing or peptide processing are needed to validate our hypothesis.

The characteristics of DEPs in infected maize showed specificity in protease cleavage sites and isoelectric point range. Most of the DEPs were internally from precursor proteins, whereas some were from the C-terminal, and approximately 1% were from N-terminal. The functions of the precursor proteins mostly involved metabolic processes, photosynthesis, and carbohydrate metabolic processes. Thus, we speculate that the functions of these DEPs could be the same or different from those of their prerequisite proteins. Further studies on the functions of the DEPs identified in this study will provide new insights into understanding the mechanisms of maize response to SCLB.

In the present study, we found that a peptide SRINPLVRLK was present in both groups simultaneously. The peptide is located at 143-152 of maize extracellular ribonuclease LE (ID: B6SSH9). B6SSH9 was the most common precursor protein in both cases, but it has not been manually annotated in Swiss-Port database. We predicted the secondary structure of B6SSH9 (Figure 6c) using PSIPRED software [52]. The results show that another peptide EKDYFETALSFR formed a helix in B6SSH9. These findings indicate that these peptides play a significant role in maize response to SCLB infection, further suggesting that precursor proteins may have similar functions in maize SCLB resistance.

Further bioinformatic analysis of the 262 common DEPs demonstrated that the main cellular components of precursor proteins and DEGs were photosynthesis and chloroplast thylakoid membrane, consistent with RNA-Seq results, indicating that these GO terms are crucial in SCLB resistance. KEGG enrichment of precursor proteins and DEGs showed that carbon fixation in photosynthetic organisms/biological pathway was enriched in both cases. Based on their functions, these peptides may play the same role as the precursor proteins in resisting SCLB. A peptide AYGEAANVFGKTKKNTD from the oxygen-evolving enhancer protein 2-1 chloroplast increased in RNA-Seq (NM_001323898.1) but decreased in peptidome results. Therefore, more studies are needed to determine whether these peptides play the same role as their precursor proteins in responding to *B. maydis* infection.

## 4. Materials and Methods

### 4.1. Plant Materials and B. maydis Inoculation

Maize (Chang 7-2 variety) seeds were obtained from the National Engineering Laboratory of Crop Resistance Breeding, Anhui Agricultural University, China (31.5° N 117.3° E). The seeds were surface-sterilized by soaking in 70% ethanol for 3 min, then soaking in 7% NaClO for 20 min. The seeds were washed with sterilized distilled water three times after ethanol treatment and at least five times after NaClO treatment. Afterward, the seeds were germinated in a growth chamber at 22 ± 0.5 °C and 60% humidity under 16/8 h light/dark, respectively. After germination, the seedlings were transplanted into nutrient soil and cultured in a greenhouse at 28 ± 1 °C under a cycle of 16/8 h day/night, respectively, for 21 days.

Two culture media were used to culture *B. maydis*. The first was potato dextrose agar (PDA) medium; 200 g of sliced peeled potato was boiled in 1 L of distilled water for about 1 h and filtered through 2-4 layers of gauze to save effluent (potato infusion). Then, 20 g/L of dextrose and 10-20 g/L of gar were added to the filtrate, and the volume was adjusted to 1 L with distilled water. The medium was sterilized using an autoclave at 121 °C for 20 min. The second was corn kernel medium; corn kernel was soaked in water for 24 h, the surface water was absorbed with filter paper, and then sub-packed into tissue culture bottles (1/2 volume), sterilized at 121 °C for 1 h, and left to cool at room temperature until use.

Pure virulent strain of *B. maydis* was provided by Prof. Ding (School of Plant Protection, Anhui Agricultural University, Hefei, China). The fungus was activated and cultured in PDA medium in a Petri dish. Afterward, mycelia were transferred from the Petri dishes to corn kernel medium and cultured in the dark at 28 °C for 10 days. Then, the spores were induced by ultraviolet light and washed with 0.05% Tween-20 aqueous solution. Spore suspension (1 × 10^5^ U/mL) of *B. maydis* was evenly sprayed on corn leaves at the 6-7 leaf stage in the evening. After inoculation, the plants were bagged for 24 h to keep moisture. Leaves were collected at day 1 and day 5, respectively, and those without infection were considered the control group and those with infection were considered the infected group.

### 4.2. Measurement of ROS Accumulation, MDA Content, and Antioxidant Enzyme Activities

Superoxide anion radical (O_2_^−^) accumulation in the leaves was determined by DAB and NBT staining as described previously [53,54]. MDA content and antioxidant enzyme activities were measured before and after pathogen infection, as described in a previous study with minor modification [55,56]. Briefly, the leaf samples were homogenized in phosphate-buffered solution (pH 7.8), then the supernatant was incubated with 5% TBA at 100 °C for 10 min, and the absorbance was measured at 600 and 532 nm. A decrease in NBT absorbance at 560 nm was used to assess SOD activity, whereas the disappearance of H_2_O_2_ at 240 nm was used to determine CAT activity. The oxidation of guaiacol was used to determine POD activity.

### 4.3. Total RNA Isolation, mRNA Library Construction, and Sequencing

Two biological replicates were performed for RNA-seq. We named the uninfected maize leaves at day 5 CK-5d-1 and CK-5d-2, while the infected maize leaves at day 5 were named SCLB-5d-1 and SCLB-5d-2. RNA-seq libraries were prepared and sequenced by Applied Protein Technology Co., Ltd. (Shanghai, China). RNA-seq analysis of the constructed library was performed using the Illumina HiSeq 2000 sequencing system with an Agilent 2100 Bioanalyzer and 2100 RNA Nano 6000 Assay Kit (Agilent Technologies, Santa Clara, CA, USA). The quality of raw readings was evaluated using FastQC [57] software (v0.11.3) (Babraham Bioinformatics, Cambridge, UK). Trimmomatic [58] was used to eliminate reads containing adapter or poly-N from the original data to obtain clean reads. The clean reads were aligned with the B73 maize genome (https://maizegdb.org/B73_RefGen_v4, accessed on 15 April 2021) using Hisat2 [59]. StringTie software (v1.0.4) [60] was used to assemble novel transcripts and multiple transcripts produced by alternative splicing. Gene expression was assessed by measuring fragments per kilobase of transcript per million mapped reads (FPKM). Differentially expressed genes (DEGs) were identified using DESeq2 [61]. The false discovery rate (FDR) was used to determine the threshold of the *p*-value in multiple tests. The FDR-adjusted *p*-value ≤ 0.01 and the |*log_2_* fold change| ≥ 2 were taken as the threshold to judge the significance of gene expression difference. The transcriptome data were uploaded to NCBI database (SRR16934612-SRR16934615).

### 4.4. Peptide Extraction and Tandem Mass Tag (TMT) Labeling

The uninfected, as well as the infected, maize leaves at day 1 and day 5 were named CK-1d, CK-5d, SCLB-1d and SCLB-5d, respectively. Infected and uninfected maize leaves were irradiated in a conventional microwave oven set at high power for 20 s to rapidly increase the leaf temperature to 80 °C. Afterward, the leaves were ground in liquid nitrogen and suspended in 1.5 times volume of precooled acetone (20 °C), containing 10% (*v*/*v*) TCA and 0.07% (*v*/*v*) 2-mercaptoethanol. After precipitation at −20 °C for 1 h, the collected proteins were centrifuged at 10,000 rpm at 4 °C for 10 min. The pellets were washed twice with cold acetone containing 0.07% (*v*/*v*) 2-mercaptoethanol. The protein pellets were lyophilized and kept at −80 °C or immediately extracted with protein extraction buffer containing 8 M urea. The protein extracts were centrifuged at 12,000 rpm for 30 min at 4 °C. The protein content in the supernatant was quantified using a protein assay BCA kit (Solarbio, Beijing, China) according to the manufacturer’s instructions. The supernatant was filtered through a Microcon^®^ YM-10 centrifugal filter unit (Millipore, Billerica, MA, USA) to remove the proteins with a molecular weight greater than 10 kDa. The peptide filtrate was desalted and concentrated on a PierceTM C18 spin column (ThermoFisher Scientific, Waltham, MA, USA) according to the instructions of the manufacturer. TMTsixplex^TM^ Label Reagent Set was purchased from ThermoFisher Scientific (Waltham, MA, USA). Each TMT labeling reagent was dissolved in 42 μL of ACN, then 2.5 μL was added to each sample and incubated at room temperature for 1 h. Three sets of biological replicate peptide samples were marked with TMT 6-plex labeling reagents according to the manufacturer’s instructions and named S1, S2, and S3. After the reaction, 5% hydroxylamine of the same volume was added to remove the TEAB nonspecifically bound to Tyr residues and quench the reaction. The labeled samples were combined, desalted, dried, and stored at −80 °C. Three biological replicates were performed.

### 4.5. Chromatography and MS/MS Analysis

Labeled peptide samples of infected and uninfected maize leaves were quantified on an EASY-nLC 1200, coupled with a Thermo Q Exactive Orbitrap Mass Spectrometer (ThermoFisher Scientific, Waltham, MA, USA). First, the peptide samples were dissolved in 0.1% formic acid and concentrated on an Acclaim Pep-Map100 C18 trap column (2 cm × 100 μm i.d., 5 μm, 300 Å, ThemoFisher Scientific, Waltham, MA, USA). Then, on an Acclaim Pep-Map RSLC C18 analytical column (150 mm × 50 μm i.d., 2 μm, 100 Å, ThemoFisher Scientific, Waltham, MA, USA), the peptides were continuously separated with a gradient elution profile. The gradient profile started from 3% to 8% solvent B within 2 min, then increased to 8% to 28% solvent B within 90 min, followed by 28% to 44% solvent B from 92-110 min, 44% to 99% solvent B from 110–112 min, and kept for 8 min at a flow rate of 3.0 μL/min. Solvent A contained 0.1% formic acid, whereas solvent B was acetonitrile with 0.1% formic acid.

The orbitrap fusion hybrid mass spectrometer was operated in high-energy collision dissociation (HCD) under the data-dependent acquisition mode. The mass spectrometer was obtained in the positive ionization mode with a spray voltage of 2.2 kV, capillary temperature of 275 °C, m/z range of 300 to 1800, and resolution of 240,000. The duration of MS survey and MS/MS accumulation were 1 and 2 s, respectively. The first six signals with the highest intense ion (>5 × 10^4^) in the collected MS spectra were fragmented to create the subsequent MS/MS spectra. All MS/MS images were collected using high-energy collision cracking set at 38 eV with a resolution of 30,000. The MS/MS resolution was set to 30 k, the automatic gain control was set to 5e^5^, and the maximum ion accumulation time was set to 60 ms.

### 4.6. Database Search and Peptide Identification

Multiple strategies were employed to identify peptides. Proteome Discover (ThermoFisher version 2.1) was used to identify peptides from LC-MS/MS data. Enzyme specificity was set to unspecific. The mass tolerance for fragments and peptides were 20 ppm and 0.02 Da, respectively. The sequences were searched against the *Zea mays* sequences in the UniProt database (http://www.uniprot.org/, accessed on 2 March 2021, UPID: UP000007305) and concatenated to the database of proteomics research. The fixed modifications were carbamidomethyl-Cys, 6-plex TMT at the N-termini and Lys-6-plex TMT, but Met oxidation was variable. Each peptide quantification value was exported to an Excel output file, and the average peptide ratios or fold change (FC = treatment/control) were determined by dividing the quantification value of each peptide in the treated samples by the quantification value of control samples. Differentially expressed peptides (DEPs) represented peptides with FC ≥ 1.25 (up-regulated) or ≤0.8 (down-regulated), along with significantly different abundances (*p*-value < 0.05). The peptidomics data were uploaded to iProX database (iProX ID: IPX0005992000).

### 4.7. Functional Enrichment Analysis

To establish the cellular components, molecular functions, and biological processes involved in DEGs, we utilized the web-based Omicsbean program [62] (http://www.omicsbean.cn/ accessed on 2 March 2021) to classify the function of Gene Ontology (GO) [63] and the Kyoto Encyclopedia of Genes and Genomes (KEGG) [64]. The GO database (http://www.geneontology.org/ accessed on 2 March 2021) was utilized to annotate DEGs and DEPs. KEGG is a route database for systematic analysis of gene functions (http://www.genome.jp/kegg/ accessed on 2 March 2021). KEGG pathways are classified as follows: A is for Metabolism; B is for Genetic Information Processing; C is for Environmental Information Processing; D is for Cellular Processes; and E is for Organismal Systems. KEGG pathway enrichment analysis adopts the same hypergeometric method as GO enrichment analysis. During analysis, the rowttest function performed a *t*-test for each gene using genefilter, *p-*value was set to <0.05 and calculated using Fisher’s exact test with a hypergeometric algorithm, and the ‘Benjamini–Hochberg’ method for multiple test correction were used for *p-*value adjustment analysis. The adjusted *p-*value < 0.05 was considered statistically significant. Proteins and genes with apparent expression changes were enriched for GO and KEGG pathways, and their functions were identified.

## 5. Conclusions

This study identified DEPs and DEGs between *B. maydis*-infected maize and the corresponding uninfected maize, enabling systematic analysis of genes and peptides associated with SCLB resistance in maize. These findings provide new perspectives toward understanding the molecular mechanisms underlying SCLB resistance. Further studies are needed to discover the functions of each peptide in response to *B. maydis* infection in maize.

## Figures and Tables

**Table 1:** Overview of the sequence assembly after Illumina sequencing.

Samples	Raw Reads	Clean Reads	Q20 (%)	Q30 (%)	GC (%)
CK-5d-1	52040214	50857364	98.26	94.87	55.07
CK-5d-2	50225462	48705260	98.08	94.43	54.84
SCLB-5d-1	50900144	49825876	98.34	95.07	55.36
SCLB-5d-2	53742962	52147524	98.26	94.90	57.64

**Table 2:** Ten common DEPs found in maize leaves on day 1 after infection with *Bipolaris maydis*.

Peptide Sequence	Accession	Precursor Protein	Positions	MH + [Da]	FC
SRINPLVRLK	B6SSH9	Extracellular ribonuclease LE	(143–152)	1654.089	0.22
AKGIEPDFGLYGLK	B6SSH9	Extracellular ribonuclease LE	(153–166)	2195.304	0.44
LKAKGIEPDFGLYGLK	B6SSH9	Extracellular ribonuclease LE	(151–166)	2665.646	0.48
EKDYFETALSFR	B6SSH9	Extracellular ribonuclease LE	(131–142)	1964.053	0.65
AYPTSDVVIETHKEEEL	P27787	Ferredoxin-1, chloroplastic	(131–147)	2418.28	1.32
LVLPGELAKHAVSEGTKAVTKFTSS	Q43261	Histone H2B.3 *	(214–238)	3487.071	1.44
LVLFEHFGGDPSKISF	A0A1D6JWI7	Beta-galactosidase, EC 3.2.1.23	(767–782)	2251.253	1.42
LVLLEEFGGDLPGVKLVTRTA	B6T0D0	Beta-galactosidase, EC 3.2.1.23	(703–723)	2685.596	1.40
LVLLEEFGGDLPGVKLVT	B6T0D0	Beta-galactosidase, EC 3.2.1.23	(703–720)	2357.41	2.05
GLGGLFAKKSS	B4FS10	Uncharacterized protein	(107–117)	1752.099	2.18

* Manually annotated in Swiss-Port.

**Table 3:** Twenty common DEPs found in maize leaves on day 5 after infection with *Bipolaris maydis*.

Peptide Sequence	Accession	Precursor Protein	Positions	MH + [Da]	FC
FISYVGDGFKLL	A0A1D6F9C2	Oxygen-evolving enhancer protein 2-1 chloroplastic *	(90–101)	1817.061	0.63
AYGEAANVFGKTKKNTD	A0A1D6F9C2	Oxygen-evolving enhancer protein 2-1 chloroplastic *	(73–89)	2730.56	0.62
ALGDVLAKLG	Q41048	Oxygen-evolving enhancer protein 3-1, chloroplastic *	(208–217)	1414.903	0.55
ALGDVLAKLA	A0A1D6EXK9	Oxygen-evolving enhancer protein 3-1, chloroplastic	(207–216)	1428.919	0.64
DLDHAAKIKSTPEAEKYFAATKD	A0A1D6EXK9; B6TI20	Oxygen-evolving enhancer protein 3-1, chloroplastic, OEE3	(184–206); (185–207)	3695.103	0.69
AAKLIRTQLASAK	Q2QLY5	5-methyltetrahydropteroyltriglutamate-homocysteine methyltransferase 1, EC 2.1.1.14	(754–766)	2058.337	0.51
ALAKYFIGSVL	A0A1D6L4E9	Exoglucanase1	(101–111)	1640.019	0.69
AALVSAFASKGLD	A0A1D6H658	Peroxidase, EC 1.11.1.7	(169–181)	1708.005	0.64
AASEDTSASGDELIEDLK	B6TCN7	Threonine endopeptidase	(48–65)	2309.176	0.69
SRINPLVRLK	B6SSH9	Extracellular ribonuclease LE	(143–152)	1654.089	0.72
GDDLVDVLK	B4FAJ3	Uncharacterized protein	(179–187)	1431.846	0.71
ATSTTDLPASYGVALGTGNYVVPVRLGTPAERF	A0A1D6LRY4	Microtubule-associated protein MAP65-1	(142–174)	3609.911	1.29
AQLDATYFAMEKLG	A0A1D6IE31	Glucan endo-1,3-beta-D-glucosidase, EC 3.2.1.39	(244–257)	2032.083	1.31
AVYQRSGGAPGGDADGGVDDDHDEL	B4FWJ8	Luminal-binding protein 3, BiP3 *	(639–663)	2702.213	1.34
YILATSSNGYDPNFF	B6U534	PSI-G (Photosystem I reaction center subunit V, chloroplastic)	(131–145)	1937.948	1.50
AKGIEPDFGLYGLKAITKVF	B6SSH9	Extracellular ribonuclease LE	(153–172)	3083.868	1.51
AKANSLAQLGKYTSDG	B4FTI5	Fructose-bisphosphate aldolase, chloroplastic, EC 4.1.2.13 (Chloroplastic aldolase, AldP)	(357–372)	2311.322	1.51
AKANSLAQLGKYTSDGEAAE	B4FTI5	Fructose-bisphosphate aldolase, chloroplastic, EC 4.1.2.13 (Chloroplastic aldolase, AldP)	(357–376)	2711.482	1.54
ASTEEAVEAPKGFVAPQLD	B4FAW3	Photosystem I reaction center subunit II (Photosystem I reaction center subunit II-1 chloroplastic)	(47–65)	2417.296	1.58
AVGDLAFKALTAGLGVATLY	A0A1D6FI52	Uncharacterized protein	(2–21)	2409.416	2.68

* Manually annotated in Swiss-Port.

## Data Availability

All data are displayed in the manuscript and Supplementary Materials.

## Supplementary Materials

The following supporting information can be downloaded at: https://www.mdpi.com/article/10.3390/plants12061307/s1, Table S1: List of DEGs identified in infected maize; Table S2: List of peptides identified in S1; Table S3: List of peptides identified in S2; Table S4: List of peptides identified in S3; Table S5: Identified DEPs in IF-1D; Table S6: Identified DEPs in IF-5D; Table S7: 262 common DEPs identified in IF-1D & IF-5D. Table S8: GO enrichment results of DEGs; Table S9: KEGG pathway analysis of DEGs; Table S10: GO enrichment results of DEPs; Table S11: KEGG pathway analysis of DEPs; Table S12: List of acronyms.
